# A Robotic Clamped-Kinematic System to Study Knee Ligament Injury

**DOI:** 10.1007/s10439-024-03624-8

**Published:** 2024-10-02

**Authors:** Ophelie M. Herve, Will Flanagan, Jake Kanetis, Bailey Mooney, Thomas J. Kremen, David R. McAllister, Tyler R. Clites

**Affiliations:** 1 Department of Mechanical and Aerospace Engineering, University of California, Los Angeles, CA 90095 USA; 2 Department of Orthopaedic Surgery, David Geffen School of Medicine at UCLA, Los Angeles, CA 90095 USA; 3 Department of Bioengineering, University of California, Los Angeles, CA 90095 USA

**Keywords:** Robotic manipulator, Biomechanics, Knee joint, ACL injury

## Abstract

Knee ligament injury is among the most common sports injuries and is associated with long recovery periods and low return-to-sport rates. Unfortunately, the mechanics of ligament injury are difficult to study in vivo, and computational studies provide limited insight. The objective of this study was to implement and validate a robotic system capable of reproducing natural six degree-of-freedom clamped-kinematic trajectories on human cadaver knees (meaning that positions and orientations are rigidly controlled and resultant loads are measured). To accomplish this, we leveraged the field’s recent access to high-fidelity bone kinematics from dynamic biplanar radiography (DBR), and implemented these kinematics in a coordinate frame built around the knee’s natural flexion–extension axis. We assessed our system’s capabilities in the context of ACL injury, by moving seven cadaveric knee specimens through kinematics derived from walking, running, drop jump, and ACL injury. We then used robotically simulated clinical stability tests to evaluate the hypothesis that knee stability would be only reduced by the motions intended to injure the knee. Our results show that the structural integrity of the knee was not compromised by non-injurious motions, while the injury motion produced a clinically relevant ACL injury with characteristic anterior and valgus instability. We also demonstrated that our robotic system can provide direct measurements of reaction loads during a variety of motions, and facilitate gross evaluation of ligament failure mechanisms. Clamped-kinematic robotic evaluation of cadaver knees has the potential to deepen understanding of the mechanics of knee ligament injury.

**Supplementary Information:**

The online version contains supplementary material available at 10.1007/s10439-024-03624-8.

## Introduction

Athletic injury is a common side effect of participation in sports [1–4]. Knee injuries are among the most severe of commonly occurring lower-extremity injuries, requiring 6 months absence from sport in almost 80% of patients, and reducing long-term athletic performance in approximately 78% of injured athletes [4–6]. Trauma to the knee most commonly causes soft tissue injury to one or more of the knee’s four primary ligaments [7, 8]: the anterior and posterior cruciate ligaments (ACL and PCL), which stabilize anterior and posterior translation of the tibia and resist axial tibial rotations, and the medial and lateral collateral ligaments (MCL and LCL), which stabilize medial and lateral translation of the tibia and resist varus and valgus rotation of the knee joint. Together these ligaments ensure structural integrity of the knee, such that damage to any one of these structures causes knee instability and pain [9, 10] and increases the risk of future injury or complications and osteoarthritis [11–17].

Decades of research into knee injury have provided an understanding of the general types of motions that cause damage to each knee structure [10]. Despite these efforts, it has been difficult to define the *specific* joint conditions that most commonly lead to a given injury [10, 18–21], due in large part to difficulty measuring these conditions in vivo. To state the obvious, injury cannot be replicated on a human subject in vivo in a laboratory setting, because that would require injuring the human subject. As an alternative, experimental studies of human cadavers provide a direct means of investigating injury kinematics and kinetics, because cadaveric knees can be dissected, instrumented, manipulated, and injured. Robotic manipulators have recently gained favor as a means of implementing carefully controlled motions and loads on knee specimens [22–27]. Typically these motions are simplified to represent only a small subset of the knee’s six degree-of-freedom (DOF) motion space [28–31]. Progress has been made in reproducing in vivo ovine kinematics on a parallel robotic manipulator while measuring resulting loads [32]. Other groups have implemented complex subject-specific joint kinematics derived from in vivo ovine motions onto a porcine model as a biomechanical surrogate [33–35]. In the limited cases where in vivo kinematic recordings were “replayed” on a human cadaveric knee in six kinematic DOFs, these motions had to be modified in unrealistic ways to avoid catastrophic bony collisions [36, 37], or the cadaver was dissected such that the ACL was the only structure left intact [34]. These previous studies have highlighted the potential of robotically controlled cadaver experimentation to elucidate crucial biomechanical characteristics of the knee.

It is worth noting that computational models of ligament strain can provide an alternative means of studying injury; however, these simulations are limited because they typically assume that ligaments are straight lines anchored to bone at a single point, disregarding how they wrap around surfaces in three dimensions [38–43]. The three-dimensional nuances to each structure’s path through space are of particular importance in ligament injury because the length changes that induce injury are often extremely subtle (on the mm scale) [44]. Additionally, factors such as age, anatomy, and sex differences are also difficult to capture in computational models, and yet have been proven to play a role in vulnerability to injury [45–50].

Kinematic clamping (i.e., rigidly enforcing joint kinematics and measuring reaction loads) provides one means of exploring the complex mechanics that underlie knee injury. From a high level, ligament injury can be thought of as a kinematically definite problem, because injuries are typically caused by over*strain*, which is a kinematic problem [51, 52]. Because ligaments act as passive stiffening springs, with a defined load-displacement behavior, the *kinetic* descriptors and net joint reaction forces associated with these injury scenarios are inherently and deterministically tied to the joint’s overall *kinematics*. The key exception to this framing is in the direction of joint compression, because the bones are so rigid that increasing contact force has an imperceptible (although non-zero) effect on kinematics. Due to this inherent coupling between kinematics and kinetics, there is much to be gained from studying injury by controlling kinematics and measuring resultant kinetics (i.e., a “clamped-kinematic” framework). This focus on kinematics is particularly useful in the study of injury, because the *kinetic* conditions that correspond to injury onset are extremely difficult to study *in situ* without affecting performance [53, 54]. It remains much more feasible to collect large datasets of non-invasive *kinematic* data from athletes (e.g., via video analyses [10, 20, 49]). Kinematics are also known to be important in injury; in fact, the fundamental operating principle of rigid bracing is to prevent injury by constraining the *kinematic* space of the knee bones to positions where injury is not possible [55].

A critical next step for robotic clamped-kinematic systems is reproduction of true-to-life knee kinematics, rather than highly simplified or modified motion trajectories, on human cadavers. To this end, we present a novel methodology and robotic system capable of reproducing scaled, sex-specific six-DOF kinematic trajectories on human cadaver knees. To our knowledge, this is the first system to enforce six-DOF kinematics from published human gait studies on cadaveric knee specimens, without modifications other than consistent subject-specific scaling. Our technique overcomes the challenges associated with clamped-kinematic replication of joint motion in two key ways. First, we leverage the field’s recent access to high-fidelity bone kinematics from dynamic biplane radiography (DBR), which provides far more accurate measurements of bone movement than conventional skin-based motion tracking [56, 57]. Second, we guarantee alignment between the knee’s natural flexion–extension axis and the primary rotation axis of the motion data by identifying each specimen’s natural knee flexion axis (KFA). In this first implementation of our robotic framework, we also forego dynamic compressive load control. Not enforcing compressive loads helps to stabilize the robotic controller and ensure repeatability of the kinematic trajectories. We believe it is reasonable to omit these loads in our study of ligament damage because ligaments stretch in response to loads that create gross kinematic deformation. Therefore, any load that does not create gross kinematic deformation of the knee (such as joint compression) will not affect the ligaments. Our clamped-kinematic framework is not intended to supplant existing kinetics-based approaches; indeed, our intent is to supplement these approaches with a system that allows for robust implementation of as-recorded tibiofemoral kinematics, while measuring six-DOF ligament reaction forces and moments [27, 58], ligament strain via a linear variable displacement transducer (LVDT) [59], and onset of ligament injury [33, 36, 37, 58, 60]. To validate our methodology, we also programmed our system to move the knee specimen through simulated clinical stability tests [61–65], which allows us to quantify and compare the structural laxity of the knee before and after different motions, manipulations, or interventions.

In this study, we evaluate our robotic system in the context of ACL injury. ACL injury is the most common sports-related knee injury, occurring at least twice as often as any other knee ligament injury [7, 9]. Approximately 80% of ACL injuries occur in a non-contact scenario, meaning that no external body of mass (such as another person’s body) makes contact with or exerts force on the knee [66, 67]. This type of injury typically happens during motions that involve an abrupt change in direction or put the knee at risk of misalignment, such as pivoting or landing from a jump [68–71]. Without external impact to the joint, the loading of the knee’s passive structures is fully described in the knee’s kinematics, making non-contact ACL injury an ideal candidate for our kinematically clamped system. We first show that our system can reproduce active knee motions that do not induce injury (non-injurious motions) and an ACL injury inducing motion (injurious motion) on cadaveric specimens, and compare the structural integrity of the knee before and after each set of motions. We then use data from the instrumented robotic manipulator and cadaver specimen to demonstrate how this system can be applied to study ligament strain, reaction kinetics, and mechanisms of soft tissue injury, which are elements of knee joint biomechanics that are not currently possible to reproduce in computational simulation.

## Methods

### Specimen Preparation

**Fig. 1:**
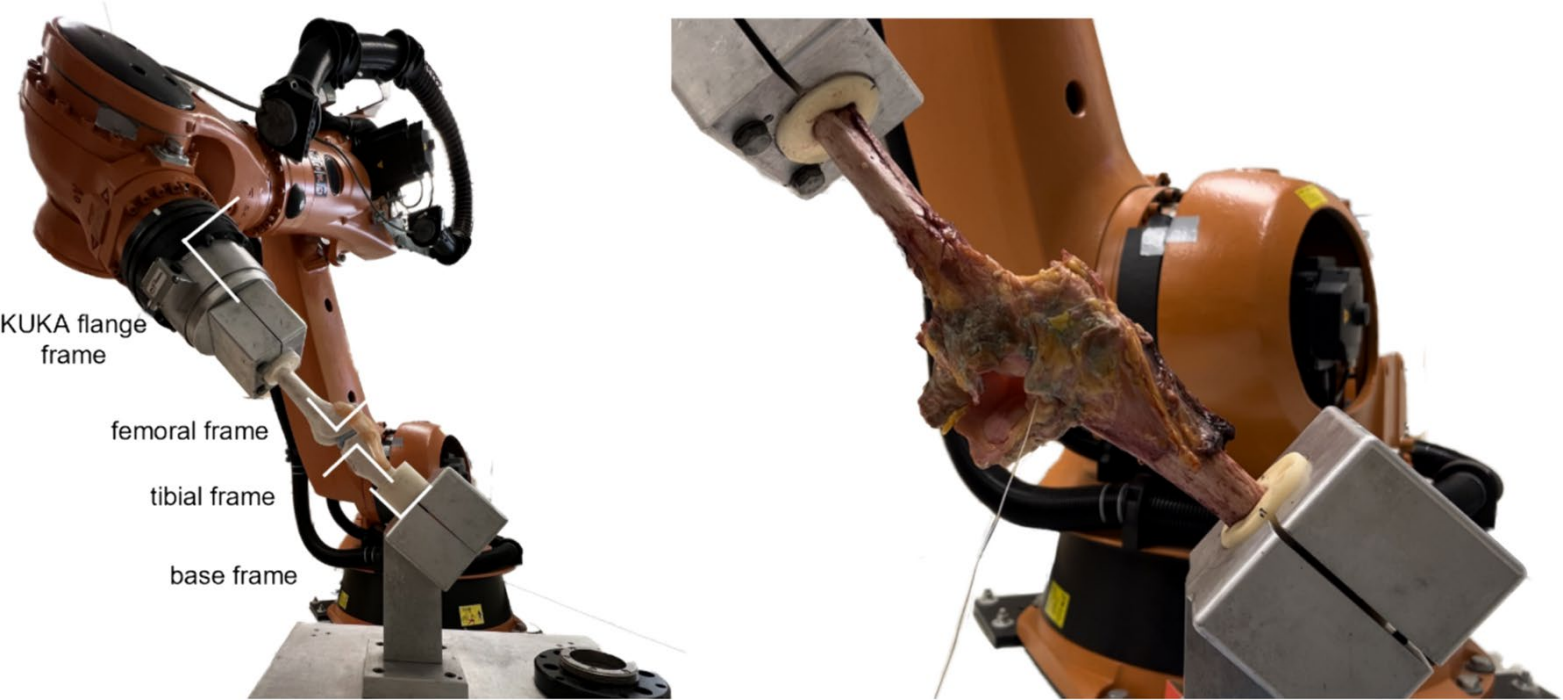
Robotic manipulator (left) with model femur in flange clamp and model tibia in base clamp. Human cadaver knee (right) in robotic manipulator with femur in flange clamp and tibia in base clamp. The LVDT is implanted onto the ACL, and its cable is seen exiting the medial parapatellar incision

Seven fresh-frozen cadaveric knee specimens (19-66 years of age, mean 33.0 years, median 30.0 years, 5 female and 2 male) were obtained from the University of California’s Donated Body Program and MTF Biologics, Inc. (Edison, NJ). Specimens were included based primarily on age and availability, and screened and excluded for prior history of knee trauma or any condition that could affect ligament integrity. Knee-en-bloc specimens were delivered with intact soft tissues from the femoral head to the ankle. First, the knees were segmented at the mid-tibial and mid-femoral level. Skin, subcutaneous fat, and all tendinous insertions into the tibia were dissected away. The femur was then stripped of all muscle and tendinous insertions except for the quadriceps tendon within 5–10 cm of its insertion into the patella and femur. We were careful to keep all extra-articular knee ligaments intact, including the superficial and deep MCL, LCL, and posterior oblique ligament. To access the joint, a medial parapatellar incision was performed and the patella was reflected laterally; at this step we confirmed grossly that each specimen had an intact native ACL and PCL. The exposed diaphyses of the femur and tibia were cleaned of all remaining tissue, including the periosteum. The proximal tibia and distal femur were aligned in cylindrical aluminum molds, and potted with polymethyl methacrylate (PMMA, Coe Tray Plastic, SPS Medical Supply Corporation, NY). The potted cylinder was sized such that one end of the knee could be rigidly secured in a pipe clamp fixed to the end effector of the 6-DOF robotic manipulator (KR210; KUKA Robotics Corp., Clinton Township, MI), and the other end in a second clamp on a rigidly grounded table (Fig. 1). All specimens were maintained frozen until needed and thawed overnight at room temperature prior to experimental sessions. Each knee was refrigerated overnight if testing days were sequential and refrozen and thawed if more than 12 hours was needed between experimental sessions, for a total of no more than three thaw-freeze cycles.

### Definition of Anatomical Coordinate Systems

The relative spatial orientations of the KUKA flange, femur, tibia, and base clamp are critical to the proper alignment of the knee in the robotic manipulator and programming of the kinematic trajectories. To identify anatomically relevant coordinate frames (Table 1) that link these components to each other, each knee was first secured in an apparatus that allowed manual flexion and extension of the knee joint about a single, fixed axis [72, 73]. This method is described in full in [72]. In summary, full extension of the knee was first defined as the equilibrium position when the femur was fixed at one end of the apparatus, the patella was facing the ground, and the tibia was hanging freely. A support table was then raised beneath the tibia in this extended position, to support the weight of the tibial pot. The position of the femoral fixture was then adjusted until flexion of the femur about the fixed axis of the test apparatus resulted in no motion of the tibia; this implied alignment between the KFA and the apparatus’ fixed bearing. A coordinate measuring machine, with a positional accuracy of 0.02 mm (Faro Gage, FARO Technologies Inc., Lake Mary, FL), was then used to digitize key anatomical landmarks.

**Table 1:** Femoral and tibial coordinate system definitions

	Femoral frame	Tibial frame	
X-axis	Perpendicular to Y-axis and Z-axis	Line connecting center of anterior and posterior aspects of tibial plateau	
Y-axis	Knee flexion axis	Perpendicular to Z-axis and X-axis	
Z-axis	Perpendicular to Y-axis, intersects center of distal femoral bone at insertion to pot	Perpendicular to X-axis, intersects center of distal tibia bone at insertion to pot	
Origin	Midpoint of knee flexion axis between most lateral and most medial points of femoral condyles	Resulting intersection point of Z-axis and X-axis	

The femoral coordinate system was built around the KFA, which we enforced mathematically to the direction of the rotational axis of the apparatus once the knee was positioned and aligned correctly. The midpoint of the line connecting the projection of the most medial and lateral points of the femoral condyles onto the KFA served as the origin of the femoral coordinate system. The long axis of the femur was defined as the line perpendicular to the KFA that also intersects the center point of the proximal femur where it enters the femoral pot. The anterior–posterior axis is the cross product of the KFA and the long axis, to guarantee frame orthogonality.

The tibial coordinate frame’s origin was defined as the average of the medial-, lateral-, anterior-, and posterior-most points of the tibial plateau. The tibial coordinate system was built around the anterior–posterior axis of the tibia, which we defined as the line connecting digitized points at the center of the anterior and posterior aspects of the tibial plateau. The tibia’s long axis was then defined as the line perpendicular to the anterior–posterior axis that intersects the center point of the proximal tibia where it enters the tibial pot. The medial–lateral axis was defined as the cross product of these two axes.

The sign convention for the femoral and tibial coordinate systems was established such that the lateral and distal directions from joint center were positive. To maintain a right-handed coordinate system, the positive direction of the anterior–posterior axis depended on “handedness” of the specimen. For right knees, the posterior direction of the femur and the anterior direction of the tibia were positive; the opposite was true for left knees. For the force-controlled stability tests, the tibia was clamped in the end effector of the robotic manipulator and the femur was clamped in the base; this arrangement directly simulates the clinical stability tests after which our experiments were modeled, in which physicians apply forces and torques to the tibia. For all kinematically clamped motions (both non-injurious and injurious), the femur was secured in the robotic manipulator and the tibia in the base, in a more anatomical orientation.

After each knee was digitized, the specimen-specific frame orientations were validated by programmatically positioning the robotic manipulator such that the knee would be oriented in its “rest” position from the digitization trials. The femur was then secured in the robotic manipulator clamp, and the tibia rested gently in the base clamp. The femoral frame was manually adjusted until the tibia remained relatively motionless while the knee was moved in flexion about the KFA. This step allowed for further refinement of the coordinate system assignment as defined on the manual KFA jig. At this point, the frames were finalized and the tibial pot was secured in the base clamp for subsequent testing.

### Clinical Stability Tests

#### Experimental Setup

Rigid enforcement of kinematic trajectories can subtly or catastrophically damage the cadaveric specimens, especially if those kinematic trajectories are even slightly inaccurate or misaligned [36]. To assess whether we only injured the knee in cases where injury was intended, and only in the ways we intended, we programmed the robotic manipulator to mimic six clinical stability tests commonly used to assess structural laxity of the knee. Each test was designed to isolate one of the primary knee ligaments (ACL, PCL, LCL, and MCL) by applying a controlled force or torque in one direction, only permitting motion along or about the axis of interest, and measuring deformation in the force-controlled direction. We designed the tests in this way to directly assess constraint of the knee in a single direction, which is the purpose of clinical stability tests [74]. The ACL and PCL were assessed by simulating clinical anterior and posterior drawer tests [62, 63, 75, 76], in which the knee is flexed to 90 degrees about the KFA and a 134 N force is applied along the tibia’s anterior–posterior axis in the anterior and posterior directions, respectively. The LCL and MCL were assessed by simulating clinical varus and valgus torque tests [64, 65], in which a 10 Nm torque is applied about the tibia’s anterior–posterior axis, with the knee at both full extension and 30 degrees of flexion, in the varus and valgus directions. Note that for the oldest specimen only, the forces and moments were adjusted to 90 N and 8 Nm, to ensure that the specimen was not accidentally injured by the stability tests. One other specimen was loose prior to testing, and therefore, hit geometric hardstops on the robotic manipulator slightly prior to reaching the full target loads (stopped at 100–131 N and 8–10 Nm, depending on instance of testing).

Each test was performed twice to precondition the knee, and then thrice more during which data were collected. All six stability tests were performed prior to testing, to provide a baseline subject-specific laxity and ensure that no primary knee structures were compromised during dissection and preparation. The tests were then repeated after non-injurious motions, to assess whether these motions damaged the knee, and again after the injurious motion, to validate that we had induced only a clinically relevant ACL injury. Based on established clinical precedent [49], we deemed a 3 mm difference between the maximum deformation seen in the pre- and post-injury anterior drawer test sufficient to indicate ACL rupture.

#### Statistical analysis

A series of paired t tests was performed to compare the maximum displacement during each of the six stability tests across each pairwise comparison of the three time points (prior to testing, after non-injurious motions, after injurious motion). A Bonferroni criterion was applied to correct for the multiple comparisons, with an original threshold for significance of 0.05 (corrected threshold of 0.0028). One knee was removed from all analysis of the varus and valgus tests at 30 degrees of flexion, because inconsistency in alignment of the starting position resulted in bone impingement when torque was applied during only these two tests.

Average inter-subject translation and rotation trajectories were also calculated. The two specimens that were not subjected to the full 134 N of force were excluded from these average trajectory plots for consistency in plotting.

### Clamped-Kinematic Motions

The construction of a consistent coordinate system centered around the KFA enabled us to mathematically transform kinematic data from differing sources to reproduce the representative motions on each knee specimen (Supplemental Fig. 1). All trajectories were extracted and replicated exactly from each original source, with the exception that translations were scaled to each specimen by the width of each knee (distance between the medial and lateral femoral condyle points) divided by the average width of that measurement from the respective population (78.5 mm for women and 88.6 mm for men) [48]. For sources that reported kinematics in the commonly used Grood-Suntay joint coordinate system, these motions were replicated exactly in the original frame. To enforce these motions in the robot, it was necessary to mathematically transform the Grood-Suntay kinematic trajectory to be representative of the femur’s motion with respect to the tibia in order to present the kinematic profile in a uniform manner used to program the robot. All non-injurious motions were applied to the knee continuously and quasi-statically, at 30% of the robot’s full point-to-point speed (average translational speed across all motions was 0.15 mm/sec, and average rotational speed was 0.71 deg/sec).

#### Non-injurious Motions

**Fig. 2:**
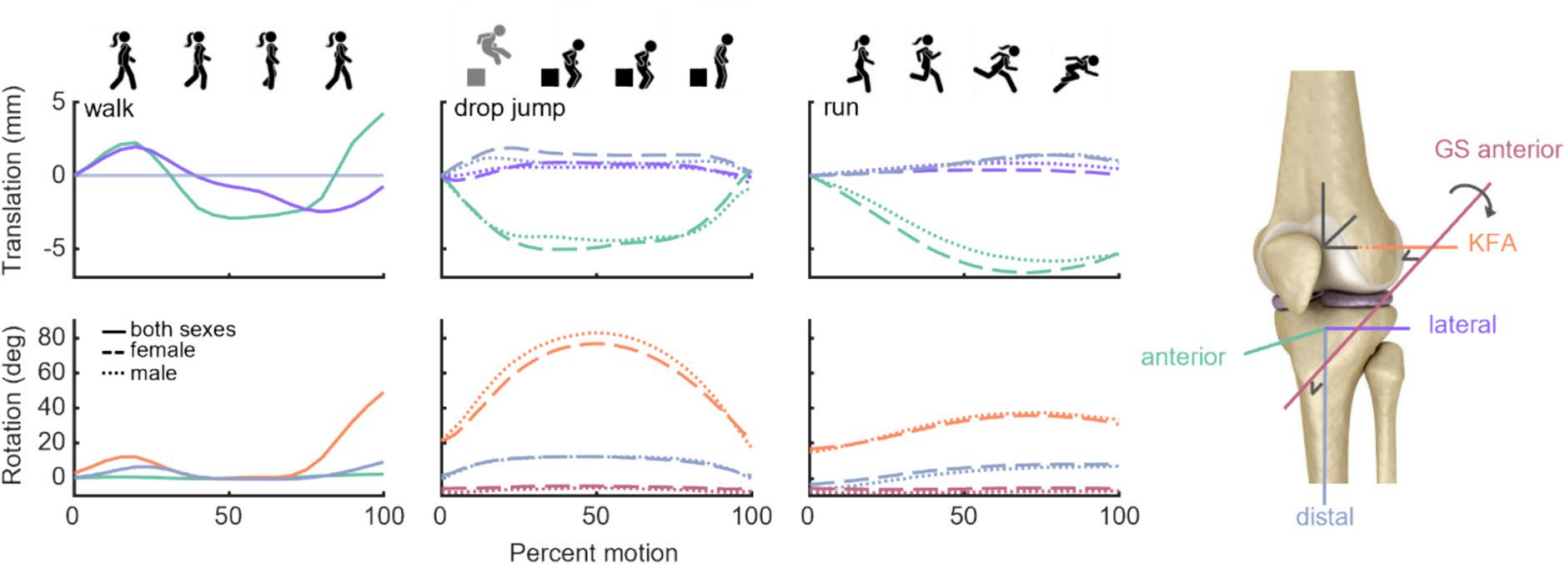
Kinematics derived from previous work depicting motion of femur with respect to axes of interest. Positive translations represent anterior, lateral, and distal motion of femur with respect to tibia along the tibia’s axes. Positive rotations represent flexion about the KFA, valgus, and external rotation of femur with respect to tibia. Valgus rotation for the walking motion [77] is defined as rotation about the tibia’s anterior axis. Valgus rotation for running and drop jump [57] are instead defined about the Grood-Suntay anterior–posterior axis [78]

Walking kinematics were extracted from a study in which DBR was used to directly measure tibiofemoral kinematics during treadmill walking [77]. Kinematics were reported in five DOFs (all but proximal-distal femoral translation); in reproducing these kinematics on our specimens, we assumed negligible translation in the proximal-distal direction. Running and drop-jump kinematics were extracted from a different study, which reports sex-specific six-DOF knee kinematics (Fig. 2), also measured via DBR [57]; these are reported in the Grood-Suntay (GS) coordinate system [78]. We interpreted all trajectories according to their published frame conventions (Fig. 2, right), and then transformed each to our anatomically relevant coordinate systems (for more, see Supplemental Methods). Each knee was preconditioned through two cycles of each motion, where each cycle consisted of moving forward from the starting position through the motion, then backward through the motion to the same starting position. Each motion was then repeated three times, while we measured ACL strain and reaction kinetics (Supplemental Video 1).

#### Injurious Motion

To injure the knee specimens, we replicated a motion that has been shown experimentally to produce isolated ACL injury [79], as a result of aggressive quadriceps loading (4500 N) at a constant 20 degrees of knee flexion. We extracted the average reaction kinematics from [79] and transformed each trajectory to our anatomical coordinate frame prior to implementation on the robotic manipulator. Each knee was then clamped into the manipulator at 20 degrees of flexion about the KFA. From this position, the knee was moved quasi-statically through the kinematic trajectory from [79], according to the following conventions: internal/external and varus/valgus rotation of the tibia was performed about the long axis and anterior–posterior axis of the tibia, respectively, in their orientations with the knee at full extension. All translations were interpreted as changes to the tibial origin from its starting position, in the tibial coordinate frame. Movement through the trajectory was paused at intervals to allow each knee to settle, and look for evidence of injury onset. Injury was definitively identified by a sudden drop in anterior joint reaction force. This large drop in force was sometimes preceded by a “crackling” sound as the individual ACL fibers began to tear. In cases when this crackling was observed prior to the definitive drop in force, the knee was left to sit for one minute to see if the injury would complete. If after one minute the drop in force was not observed in the force signal, the trajectory was moved to the next step, and this continued until injury was completed (Supplemental Video 2).

### Experimental Applications

Clamped-kinematic robotic assessment of cadaver knees provides distinct experimental capabilities that are not possible in computational modeling. Having implemented and validated our robotic pipeline, we used this pipeline to demonstrate three of these capabilities in a subset of our knee specimens.

#### Direct Measurement of Ligament Strain

Five of the seven knees were instrumented with a 3 mm LVDT (M-DVRT-3, Lord Sensing, Vermont or LVDT, Singer, Israel) to demonstrate feasibility of directly recording ACL strain during the clamped-kinematic motions. A barbed screw was secured to the housing of the sensor on either end, to allow robust fixation to the ACL. Prior to insertion in each knee, the sensor was calibrated to a known distance between barb screws. With the knee flexed and the patella reflected, the LVDT was aligned with the ACL and inserted such that the barb screws were securely implanted into the ligament. For non-injurious motions, the sensor was intentionally placed such that the sensor could be compressed and elongated without reaching the bounds of its range. For the injurious motion, the sensor was removed and re-inserted such that the barbs began closer together, to maximize the range over which the sensor could elongate. Strain for each motion was calculated with respect to the zero-force, light tension distance between the barbed screws. Due to sensor failure and migration, ACL strain data was obtained from three of the five instrumented knees during non-injurious motions, and four knees during the injurious motion. The strain signal was hard synced with the robotic manipulator’s kinematic trajectory, and strain data were digitally low pass filtered using a second-order Butterworth filter with a 15 Hz cutoff frequency (MATLAB, MathWorks, Natick, MA, USA). Note that we faced a variety of challenges when working with the LVDTs, such as difficulty in tissue implantation, high sensitivity to placement on the ligament, and sensor failure; these challenges are described in detail in the Results section.

#### Direct Measurement of Joint Reaction Loads

We used the six-axis load cell mounted to the robotic manipulator’s end effector to measure ligament reaction forces and moments during each of the non-injurious and injurious motions. Each load trajectory was then transformed into and reported in the femoral coordinate frame. Note that compressive loading was not controlled here, but the forces and moments in the other directions are useful in understanding the passive resistance of the knee joint complex to these motions. Reaction load data were filtered at 15 Hz, and peak loads were compared across activities. Reaction loads were compared across activities using a one-way ANOVA (alpha = 0.05), Bonferroni corrected to account for 6 hypothesis tests (corrected alpha = 0.0083).

#### Post-injury Analysis

Cadaver-based models provide the unique opportunity to examine the specimen after experimentation. Through gross visual and physical inspection, we qualitatively confirmed injury and classified the specific mechanism of failure for each knee. We also quantitatively assessed the integrity of each knee after injury, according to the stability tests described above.

## Results

### Validation of Experimental Pipeline

**Fig. 3:**
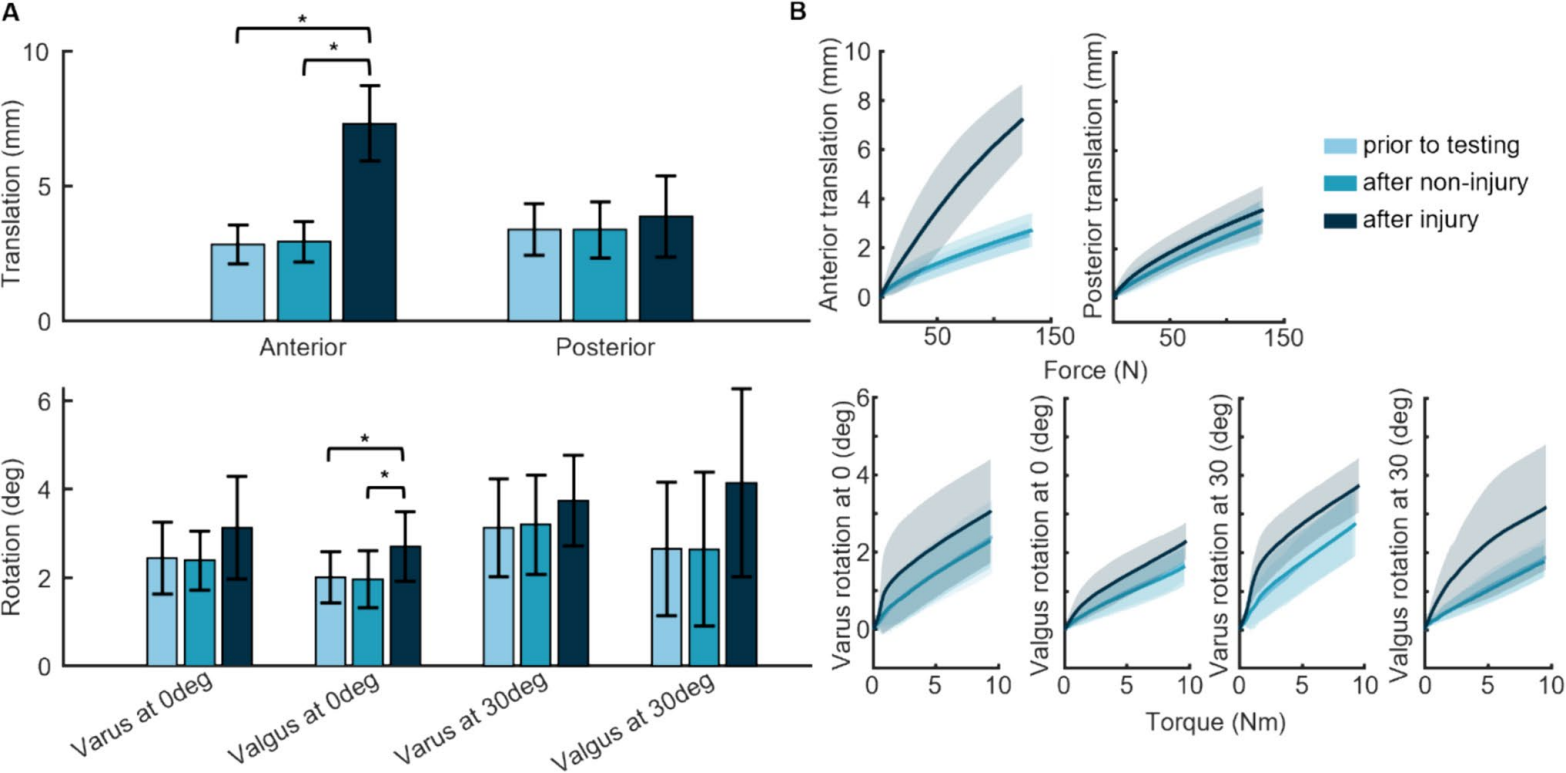
Stability test results. **A** Inter-subject average maximum translation/rotation during each of the six stability tests. Error bars show + /– 1 SD. **B** Translation and rotation trajectories during the six stability tests. Solid lines show inter-subject average, shaded regions show + /– 1 SD

The robotic manipulator replicated the desired kinematic trajectories to within 0.05 mm and 0.0025 degrees of the desired input. The stability tests showed no statistically significant difference between “prior to testing” and “after non-injurious” conditions, with a maximum difference of 0.55 mm in translation and 0.41 degree in rotation (Fig. 3A). However, the “after injurious” condition revealed a statistically significant increase in translation along the anterior–posterior axis of the tibia during the anterior drawer test (paired *t* test, *p* = 0.00003 in comparison to prior to testing and *p* = 0.000013 in comparison to after non-injurious), which is indicative of ligament damage (Fig. 3A, Supplemental Video 2). The average maximum translation after the injurious motion differed from the two prior instances of testing by more than 3 mm, which exceeds the clinical threshold for injury diagnosis. A statistically significant difference was also seen in the rotation about the anterior–posterior axis of the tibia during the valgus test at full extension (*p* = 0.0005), which may be indicative of MCL laxity [14, 80, 81]. None of the other tests showed significant change in laxity of the knee nor injury to other structures across the three testing conditions.

### Direct Measurement of ACL Strain

**Fig. 4:**
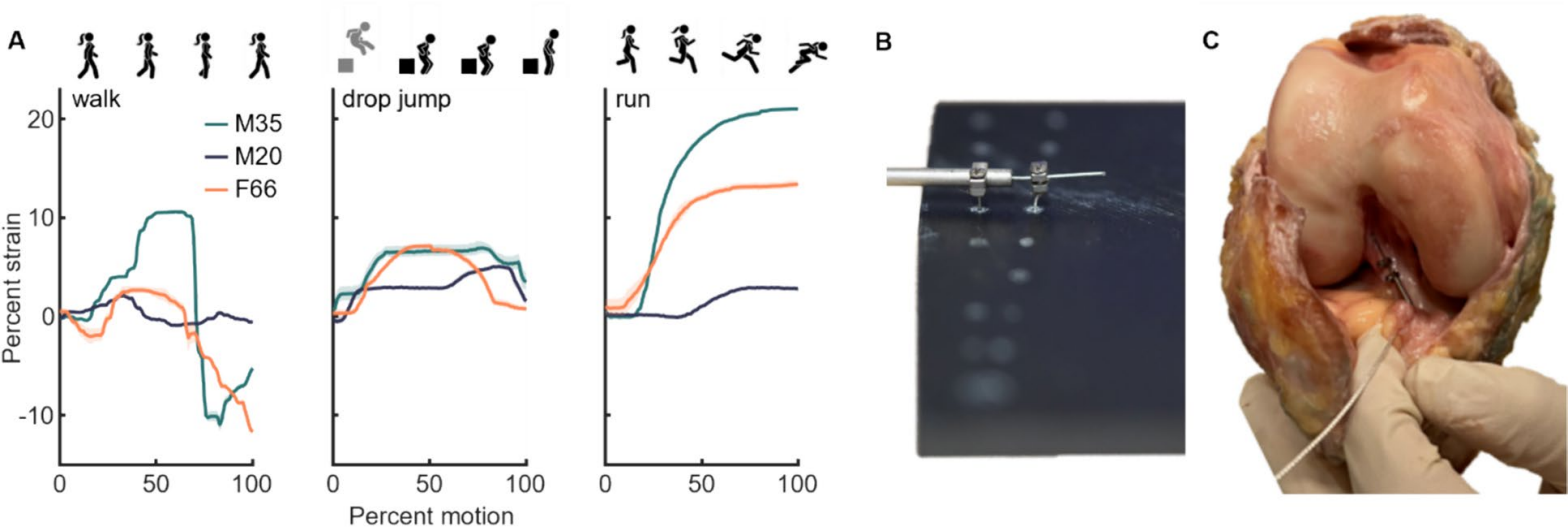
**A** ACL strain from three subjects for walking, drop jump, and running. Solid line shows intra-subject average of three trials. Shaded regions, which are difficult to see because they are small, show + /– 1 SD. **B** Calibration of LVDT where barbs are at a known distance. **C** LVDT inserted on the ACL

Strain results are consistent and repeatable for each specimen (maximum standard deviation of 1.45%), but varied greatly across specimens (Fig. 4). In the specimen with the largest strains, the LVDT measured peaks of 10.6, 6.96, and 20.98% during walking, drop jump, and running, respectively. In the specimen with the smallest strains, LVDT measured peaks of 2.16, 5.02, and 2.95%. The highest strain was experienced during running for two specimens, and during drop jump for the third. The LVDT was difficult to place, and only provided useable results in three of the five instrumented specimens; in the other two specimens, we either saw obstruction/interference from the bone, or the LVDT became dislodged.

### Joint Reaction Loads

**Fig. 5:**
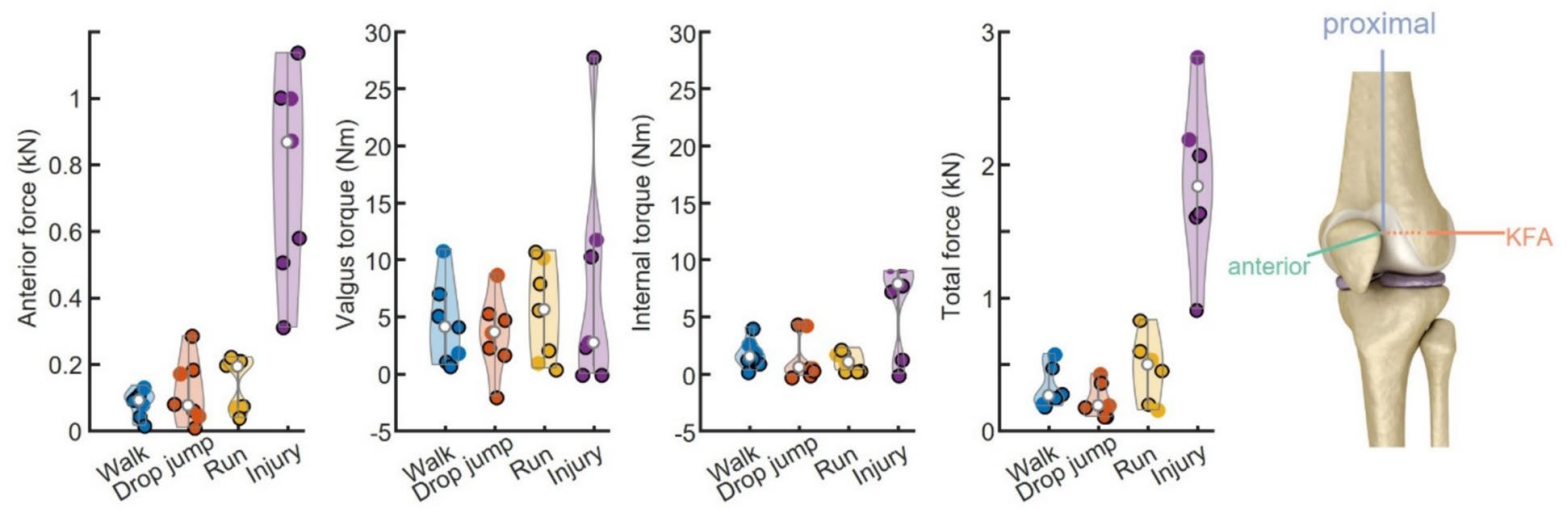
Reaction loads (left) experienced by the femur, in the femoral frame (right), during each non-injurious and injurious motion. White circles indicate mean values. Circles with black outlines are female specimens, circles without outlines are male specimens

The peak anterior forces and total forces were greater during the injurious motion in comparison to walking, drop jump, or running (one-way ANOVA, *p* = 0.00055, 0.00076, 0.0021 and *p* = 0.0016, 0.0046, 0.0015, respectively) (Fig. 5). Although the peak valgus torques were not significantly different across activities, several specimens did see relatively high peak torques. The peak internal torque was significantly higher during the injurious motion in comparison to running (*p* = 0.008) while walking and drop jump did not show significant difference (*p* = 0.0457, 0.0382). These results are consistent with current understanding of the mechanism of ACL injury. The largest peak forces among the non-injurious motions in the directions of interest were seen during running; however, drop jump showed a larger total force, indicating that the uncontrolled reaction force along the long axis of the femur was likely dominant in this case. The reaction loads varied across specimens (Supplemental Fig. 2).

### Injury Analysis

**Fig. 6:**
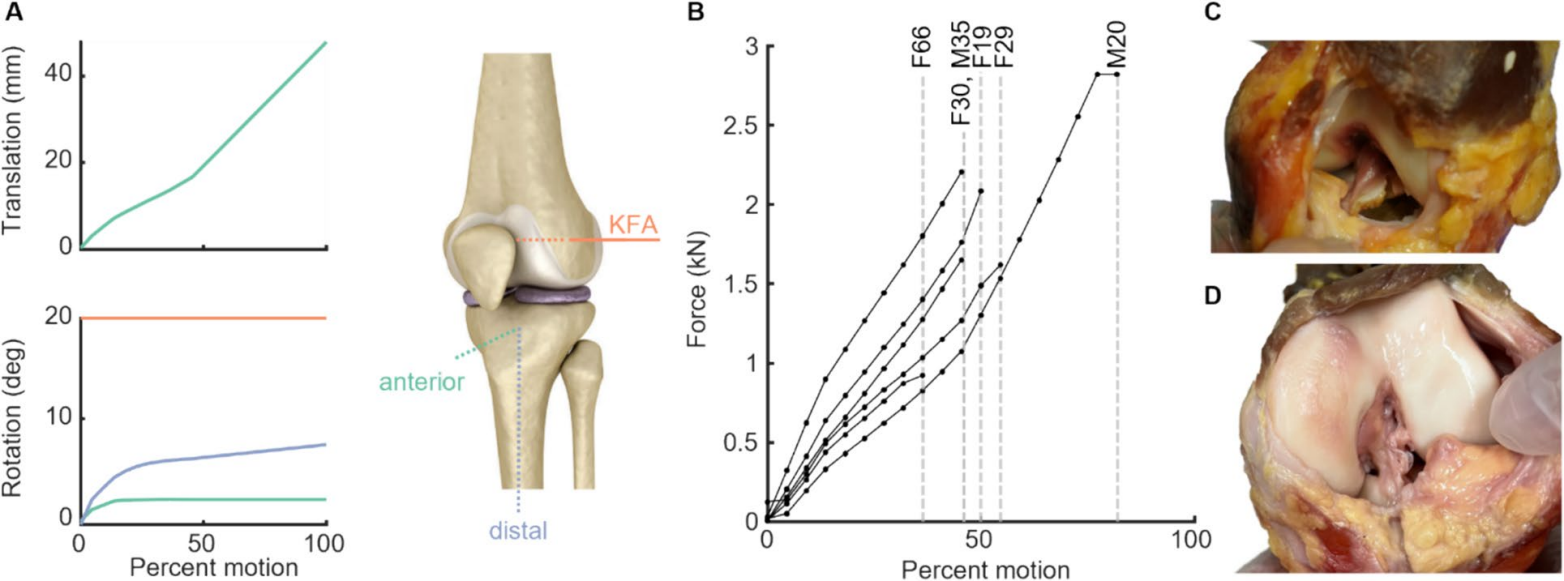
**A** Clamped-kinematic trajectory of the tibia relative to the tibial frame for the injurious motion. **B** Total reaction force in each specimen along the input trajectory. Each line ends at rupture. **C** Representative photograph of a tibial avulsion fracture. **D** Representative photograph of a femoral attachment rupture

Of the seven knee specimens, five ruptured at the femoral insertion of the ACL (Table 2). The other two ACLs failed due to bony avulsion at the tibial insertion, indicating weakness in the bone (bone failed prior to ligament). The point in the kinematic trajectory at which ACL rupture occurred varied among specimens (Fig. 6). Failure loads varied by subject, with the oldest specimen (66 years) rupturing at the lowest failure load of 930.8 N (Fig. 6). The highest total failure force, 2818.9 N, was required to rupture the youngest male specimen (20 years).

**Table 2:** Specimen demographics and failure mode.

Sex	Age	Height (m)	Mass (kg)	Type of failure
F	19	1.68	54.43	Femoral attachment rupture
M	20	1.73	78.02	Femoral attachment rupture
F	29	1.65	71.67	Tibial avulsion fracture
F	30	1.65	45.36	Tibial avulsion fracture
F	32	1.65	60.78	Femoral attachment rupture
M	35	1.88	108.86	Femoral attachment rupture
F	66	1.60	–	Femoral attachment rupture

## Discussion

In this study, we presented and validated a robotic system for high-fidelity replication of sex-specific, inter-subject average knee motions on cadaver specimens. Our results show that this system is capable of moving knees of different size, sex, and age through several active non-injurious kinematic trajectories without causing injury, and of inducing ACL injury with clinically relevant instability characteristics. We also demonstrate the potential broad utility of this system in characterizing the knee joint’s passive resistance to motion and studying with more specificity the mechanism of simulated knee injuries. Although we showed that this system is also capable of incorporating direct measurement of subject-specific localized ligament strain, it was our experience that the practical challenges associated with the LVDT are sufficient to limit the utility of the resultant data.

The results of the six stability tests confirmed that none of the seven knee specimens were injured during the non-injurious motions. To our knowledge, this is the first system to enforce inter-subject six-DOF kinematics from published human gait studies on cadaveric knee specimens, without modifications other than consistent subject-specific scaling. We attribute the success of this system in part to our use of highly accurate bone kinematics, which have only recently become available with the advent of DBR technology. Additionally, our coordinate frame definitions prioritized each subject’s empirically identified KFA; this ensured that our enforcement of knee flexion and extension, which is the dominant DOF in knee motion, did not lead to impingement or create large reaction forces within the joint. Our results also show that the injurious motion resulted in a clinically relevant ACL rupture in all seven specimens (Fig. 3). This was verified via gross inspection of each specimen, as well as in the at least 3 mm difference in the anterior drawer test before and after injury. We also observed laxity during the valgus test at full flexion, which is consistent with MCL sprain and commonly occurs in conjunction with ACL injury [81]. Anecdotally, the first author fell while skiing on a weekend break from writing this manuscript and suffered a complete ACL rupture, an MCL sprain, and a lateral meniscus tear, which mirror the pattern of injury from our experiments.

We were successfully able to measure ligament strain in a subset of the knee specimens. Installation of the LVDT onto the ACL proved quite difficult, and added several hours of experimental time to each specimen. The primary challenge was that the strain readings were extremely sensitive to placement of the sensor within the ACL, as reflected in the high inter-specimen variability in measured strains during the non-injurious motions. However, once the LVDT was in place, the strain readings across trials were remarkably consistent, such that the within-specimen trends between different motions are likely representative of the impact of those motions on the portion of the ACL to which the sensor was affixed. This provides useful information about the relative subject-specific ACL strain induced by each motion. However, based on the challenges we faced regarding sensitivity to placement and difficulty of implantation, we suggest that LVDT implantation should not be the primary source of intra-subject characterization of *in situ* ligament strain. Additionally, repeated use of the sensor during the traumatic ACL injury trials caused breakage in two different sensors, which limited our ability to collect strain data from all specimens.

Our assessment of ligament kinetics proved instructive in understanding the relative stress placed on the knee ligaments by the different motions. As expected, the injurious motion saw higher anterior force, internal torque, and total force than the non-injurious motions (Fig. 5). This is consistent with our fundamental understanding of the loading conditions that lead to ACL injury. Interestingly, the injurious motion did not induce higher valgus moments than the non-injurious motions, even though the injurious motion caused some laxity in the valgus direction. It is also important to note in interpreting these kinetic results that we did not enforce joint contact forces or intentionally place the knee in compression, because our goal was to understand ligament strain and not reaction forces from bone contact. This “kinematics-only” framework dramatically simplified the control task, but also means that the measured reaction forces in the compression direction are not truly representative of joint reaction forces that result from contact during the tibia and femur during these motions. However, all reaction forces that result from tension in the ligaments are likely representative of the knee’s quasi-static passive resistance to these motions.

**Fig. 7:**
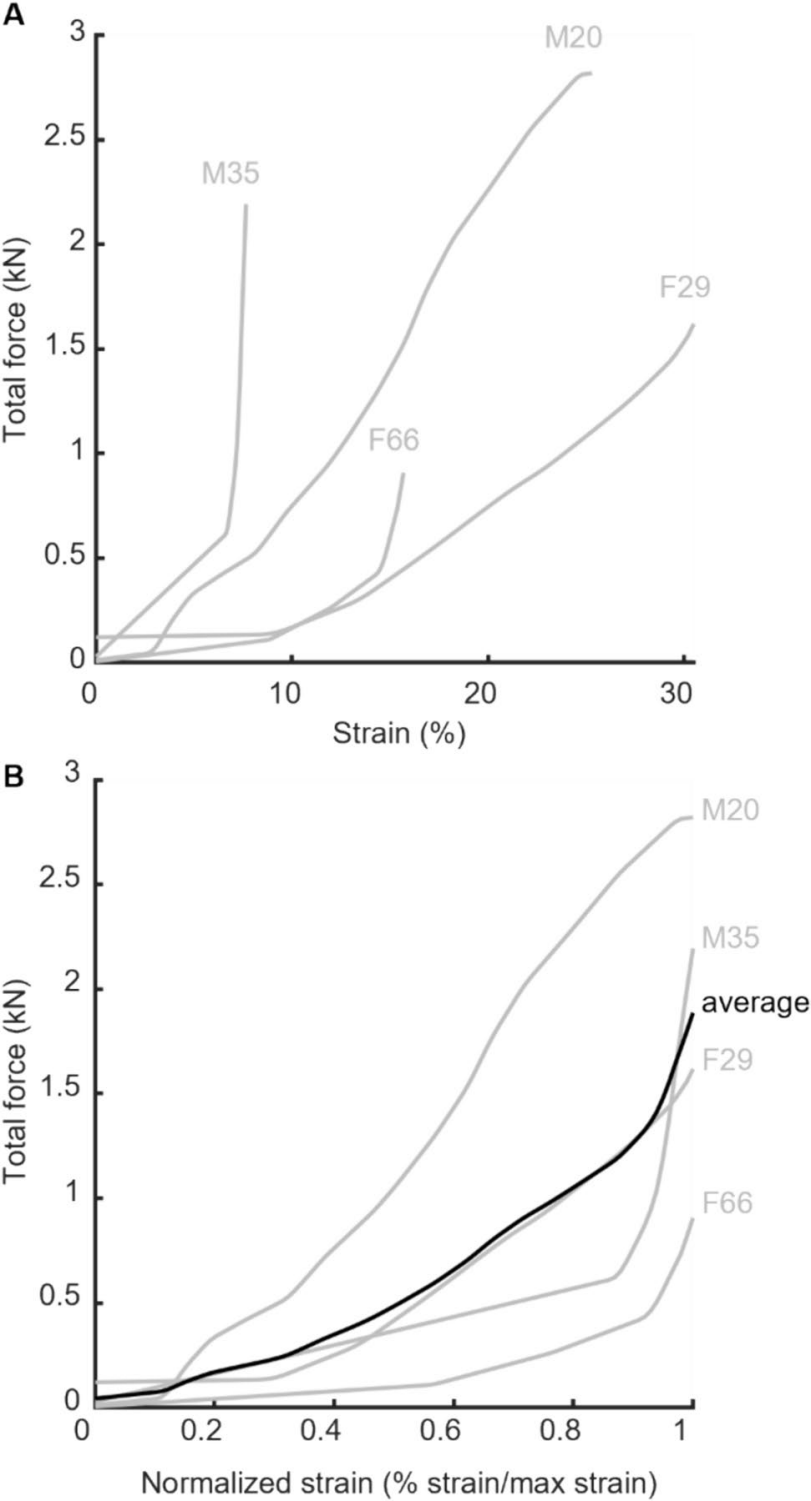
**A** Total reaction force vs percent strain. **B** Total reaction force vs percent strain for each specimen, normalized to that specimen’s maximum strain. Black line shows the average of the four specimens

A key contribution of this work is the value of our system in deepening understanding of the mechanisms that underlie ligament injury. We approach these questions from a clamped-kinematic perspective, which means that the results of our experiments tell us about the loads associated with different motions, rather than the motions induced by a given loading trajectory. We saw high variation in failure loads across subjects, and noted that the older specimens tended to fail at lower forces (Fig 6). Two male specimens also failed at higher forces than all of the female specimens. We saw differences in strain at failure as well, with two male specimens rupturing at higher strains than their female counterparts (Fig. 7). Although the sample size in this tool-validation study is not sufficient to reliably assess differences with age and sex, our results highlight the potential of this system to test hypotheses in future work related to the changes in relative susceptibility to ACL injury as a function of sex and age.

We have already noted several limitations of this study, primarily related to the challenges of LVDT placement and implantation. Most notably, we were unable to collect strain data on all of the specimens due to challenges with the sensor, namely impingement, breakage, and inability to find a suitable replacement after failure. One other consistent challenge was in bending of the bone specimens during the exceptionally high-load injurious motions. As such, we expect that the position of the femoral pot during the failure trials—which we controlled to match the failure kinematics from Ref. [79]—was not representative of the translation of the femur at the ACL insertion. For this reason, we did not use these kinematics to calculate an origin-to-insertion “global ACL strain,” as has been done in other studies [82]. Another result of this is that the kinematic trajectory of the pot may give the appearance of failure at larger translations and rotations for some knees, without these the translations and reflections necessarily being reflected at the joint center. This could be corrected by adding a motion-capture system in parallel with the robotic manipulator, to measure displacement at the joint center. Another potential limitation of the study is that all of our analysis was conducted under quasi-static loading, and so do not capture the dynamic behaviors of these ligaments.

In conclusion, we have described and validated a robotic system capable of moving a cadaveric knee specimen through six-DOF motions without causing unintended injury. We also demonstrated that this system can induce clinically relevant ligament injuries, and quantify the impact of those injuries on knee stability. Our results highlight the potential of this system to deepen understanding of injury mechanisms in ways that are not possible with in vivo studies or computational simulations. In the future, this system could be applied to evaluate sex- and age- specific susceptibility to injury, more clearly define the kinematic spaces in which injury is likely to occur, and assess the potential value of braces or other devices in preventing injury.

## Supplementary Information

Below is the link to the electronic supplementary material.

Supplementary file1 (MP4 29109 KB)

Supplementary file2 (MP4 10060 KB)
